# Electroacupuncture pretreatment induces tolerance against focal cerebral ischemia through activation of canonical Notch pathway

**DOI:** 10.1186/1471-2202-13-111

**Published:** 2012-09-19

**Authors:** Yu Zhao, Xiyao Chen, Lei Ma, Zhiyi Zuo, Zhenghua Zhu, Xiaoling Zhu, Qiang Wang, Ertao He, Lize Xiong, Jianming Pei, Lixian Xu, Lihong Hou, Shaoyang Chen

**Affiliations:** 1 Department of Anesthesiology, Xijing Hospital, Fourth Military Medical University, Xi'an 710032, China; 2 Department of Physiology, Fourth Military Medical University, Xi'an, 710032, China; 3 Department of Anesthesiology, University of Virginia, Charlottesville, VA, USA; 4 Department of Anesthesiology, Stomatology Hospital, Fourth Military Medical University, Xi'an, 710032, China

**Keywords:** Electroacupuncture, Pretreatment, Cerebral ischemia, Notch pathway

## Abstract

**Background:**

Electroacupuncture (EA) pretreatment can induce the tolerance against focal cerebral ischemia. However, the underlying mechanisms have not been fully understood. Emerging evidences suggest that canonical Notch signaling may be involved in ischemic brain injury. In the present study, we tested the hypothesis that EA pretreatment**-**induced tolerance against focal cerebral ischemia is mediated by Notch signaling.

**Results:**

EA pretreatment significantly enhanced Notch1, Notch4 and Jag1 gene transcriptions in the striatum, except Notch1 intracellular domain level, which could be increased evidently by ischemia. After ischemia and reperfusion, Hes1 mRNA and Notch1 intracellular domain level in ischemic striatum in EA pretreatment group were increased and reached the peak at 2 h and 24 h, respectively, which were both earlier than the peak achieved in control group. Intraventricular injection with the γ-secretase inhibitor MW167 attenuated the neuroprotective effect of EA pretreatment.

**Conclusions:**

EA pretreatment induces the tolerance against focal cerebral ischemia through activation of canonical Notch pathway.

## Background

Notch is a cell-surface receptor. Binding of Notch to its ligands, such as Jagged and Delta-like, results in two-step proteolytic cleavages of Notch receptor. The second cleavage accomplished by presenilin-1 and γ-secretase enzyme complex releases Notch intracellular domain (NICD) that translocates to the nucleus where it regulates transcription of the Notch tar*g*et genes, such as Hes family. This process is the canonical Notch signaling [1,2]. NICD can also induce the expression of Hes genes through the activation of cytoplasmic signals [3]. Traditionally, Notch signaling is implicated in neural development, including the maintenance of self-renewal potential in stem cells, binary cell-fate determination in progenitor cells and induction of terminal differentiation in proliferating cells [1,2,4]. Accumulating evidence indicates that canonical Notch pathway can also be activated in the adult brain subjected to stroke and traumatic brain injury [5-7]. This activation is vital in the regulation of ischemic cerebral damage, adult neurogenesis and regenerative responses after ischemia and reperfusion. However, it is still controversial whether the activated canonical Notch signaling is beneficial to the ischemic cerebral tissues.

Our previous studies have demonstrated that pretreatment with electroacupuncture (EA) at Baihui acupoint could induce two phases of tolerance to focal cerebral ischemia, a rapid tolerance occurred at 2 h after EA and a delayed tolerance occurred at 24 h after EA [8,9]. We further elucidate the adenosine, endocannabinoid and cannabinoid receptor type 1(CB1) are involved in the mechanism underlying rapid ischemic tolerance [8,10], while cannabinoid receptor 2 (CB2) is responsible for the induction of delayed tolerance [11]. Identifying the signals that mediate the neuroprotection of EA pretreatment is fundamental to reveal novel therapeutic targets. Thus, we hypothesized that Notch signaling is involved in the EA pretreatment-induced tolerance against focal cerebral ischemia. The present study was designed to testify this hypothesis in the rat subjected focal cerebral ischemia.

## Results

### EA pretreatment significantly increased Notch1, Notch4 and Jag1 mRNA in the striatum before ischemia

**Figure 1:**
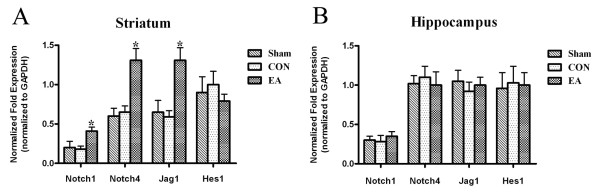
**The bar graph shows quantification of the real-time PCR analysis in the striatum and hippocampus (n = 3).** (**A**) EA pretreatment significantly up-regulated the Notch1, Notch4 and Jag1 mRNA expressions in striatum before MCAO. (**B**) There were no significant differences of mRNA expressions of Notch1, Notch4, Jag1 and Hes1 among three groups in hippocampus before MCAO. (*P < 0.05 EA vs. CON).

Real-time RT-PCR analysis for Notch1, Notch4, Jag1 and Hes1 genes, which are major Notch pathway components in the central nervous system, in striatum and hippocampus before MCAO showed that the Notch1, Notch4 and Jag1 mRNA in striatum in EA group was up-regulated compared with CON group. However, the Hes1 mRNA was not changed (Figure1A). There were no significant differences between EA and CON groups in the mRNA level of the above genes in hippocampus (Figure1B).

### EA pretreatment significantly enhanced Notch1 NICD level and Hes1 mRNA in the striatum at the early stage of reperfusion

**Figure 2:**
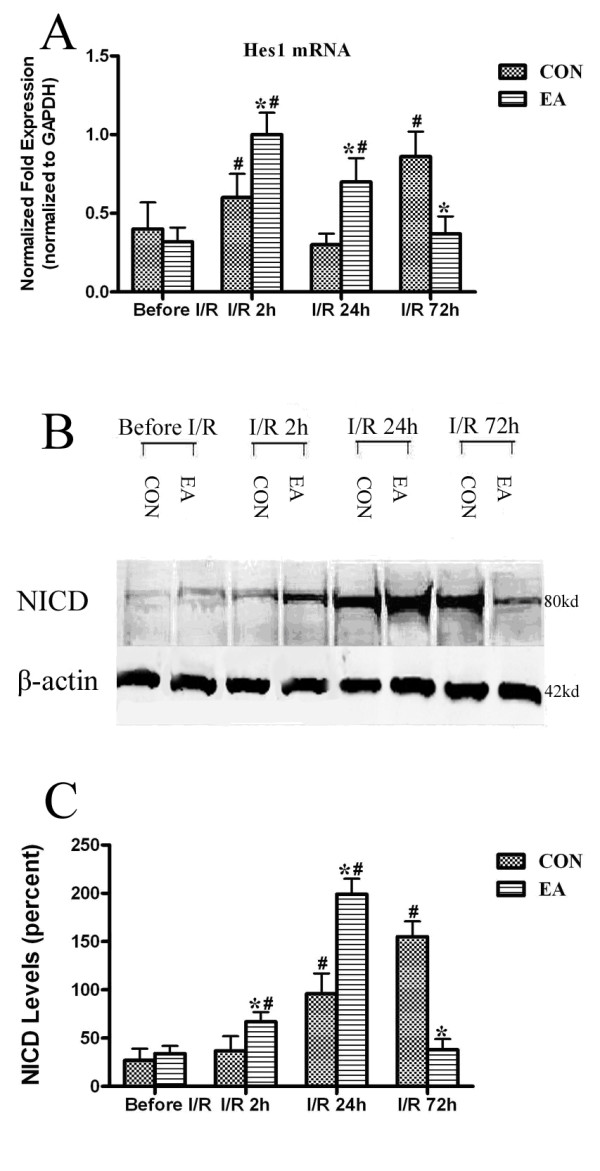
**The quantification analysis of Hes1 mRNA and Notch1 NICD protein.** (**A**) The quantification of the real-time PCR analysis of Hes1 mRNA in the striatum of rat brain compared with GAPDH respectively in CON and EA Before I/R (lane 1, 2), CON and EA I/R2h (lane 3, 4), CON and EA I/R24h (lane 5, 6), CON and EA I/R72h (lane 7, 8) groups. (**B**) Representative Western blot bands of Notch1 NICD (80 kDa) expression obtained from rats in CON and EA Before I/R (lane 1, 2), CON and EA I/R2h (lane 3, 4), CON and EA I/R24h (lane 5, 6), CON and EA I/R72h (lane 7, 8) groups. (**C**) The bar graph shows quantification of the Western blot analysis for the Notch1 NICD protein compared with β-actin. Pretreatment with EA significantly up-regulated the Hes1 mRNA and Notch1 NICD protein expressions at 2 and 24 h after the end of ischemia/reperfusion. While the expressions of Hes1 mRNA and Notch1 NICD protein of EA group were down-regulated at 72 h compared to CON group (*P < 0.05 vs. CON of the same time point, # *P* < 0.05 vs. before I/R of the same group).

Real-time RT-PCR analysis for Hes1 gene in striatum after MCAO (Figure2A) showed that in EA group, Hes1 mRNA rapidly increased to the maximum at 2 h after reperfusion (*P* < 0.05 vs. before I/R), and then gradually returned to the its pre-brain ischemia level. The content of Hes1 mRNA in EA group was significantly higher than that in CON group at 2 h and 24 h after reperfusion, respectively (*P* < 0.05 vs. CON). The variation pattern of Hes1 mRNA was different in CON group. Although Hes1 mRNA level was increased at 2 h, the peak appeared at 72 h after reperfusion (*P* < 0.05 vs. before I/R).

NICD is regarded as another activation marker of Notch signaling. There are four Notch receptors in the central nervous system. Notch1 represents a major function of Notch pathway in the brain. Thus, we measured Notch1 NICD level to further evaluate the activation pattern of Notch signaling. Western blot analysis of striatum revealed that no significant difference in Notch1 NICD level was detected between EA and CON groups before the MCAO (*P* = 0.37). In EA group, Notch1 NICD level was significantly elevated 2 h after reperfusion (*P* < 0.05, vs. before I/R) and reached the maximum at 24 h (*P* < 0.05 vs. before I/R). The NICD level of EA group was significantly higher than CON group at 2 h and 24 h (*P* < 0.05 vs. CON). The Notch1 NICD of EA group at 72 h after reperfusion decreased to a level equal to that before I/R and was lower than that in CON group at the same time point. In CON group, ischemia/reperfusion itself also significantly increased Notch1 NICD level at 24 h after reperfusion (*P* < 0.05 vs. before I/R). The Notch1 NICD level was further increased at 72 h (*P* < 0.05 vs. before I/R) after reperfusion (Figure2B and Figure2C).

**Figure 3:**
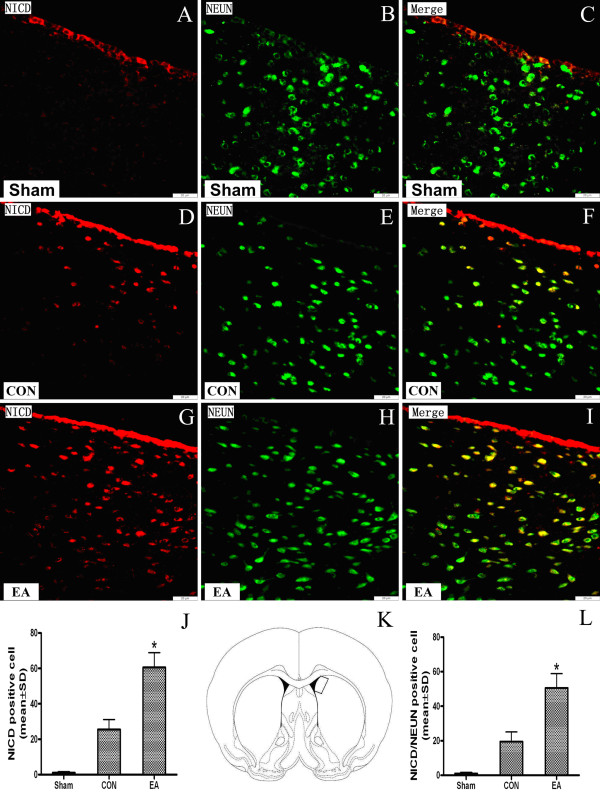
**Up-regulation of neuronal Notch1 NICD in the rat brain at 24 h after EA pretreatment.** (**A–I**) Representative double immunofluorescence staining of Notch1 NICD cells (red) and the neuronal marker (green) NeuN (neurons) in brain sections were displayed. The Notch1 NICD immunoreactivities are increased and colocalized with the neuronal immunoreactivities 24 h after EA pretreatment. Scale bars = 20 μm. Figures **J** and **L** show quantitative data of NICD- positive and NICD/NEUN double-positive cell numbers every field in the ipsilateral striatum (n = 3) . The rectangle in **K** (bregma, 0.2 mm) shows the precise region of the rat brain where these images were taken. (*P < 0.05 EA vs. CON).

As shown in Figure3A, there were few Notch1 NICD positive cells in Sham group, while Notch1 NICD positive cells were increased in CON and EA groups (Figure3D and Figure3G). Double immunofluorescent staining showed that NICD immunoreactivity was partially localized to the nuclei of cells that were also positive for NeuN, a neuronal marker, in the ischemic striatum at 24 h after reperfusion (Figure3F and Figure3I). The quantitation result revealed that there were more Notch1 NICD positive cells in EA group than CON group, and the number of cells positive for both NICD and NEUN in EA group was also much more than that in CON group(*P* < 0.05, EA vs. CON)(Figure3J and Figure3L)

### Intraventricular injections with the γ-secretase inhibitor MW167 attenuated the neuroprotective effect of EA pretreatment

**Figure 4:**
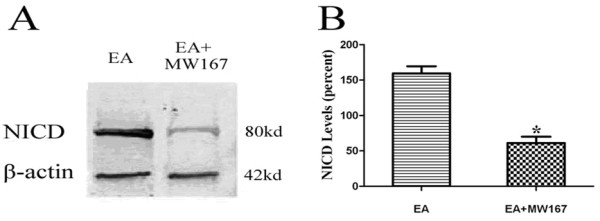
**MW167 decreases the expression of Notch1 NICD in the striatum at 24 h after I/R.** (**A**) Representative Western blot bands of Notch1 NICD (80 kDa) expression obtained from rats in EA (lane 1), EA + MW167 (lane 2) groups. (**B**) The bar graph shows quantification of the Western blot analysis for the Notch1 NICD protein compared with β-actin. (*P < 0.05 EA vs. EA + MW167).

We investigated the efficacy of MW167 in suppressing activation of Notch in the brain in vivo by measuring the level of Notch1 NICD in ischemic striatum. Western blot analysis of EA + MW167 and EA groups confirmed that 1 mM MW167 effectively inhibited Notch signal activation at 24 h after reperfusion (Figure4).

**Figure 5:**
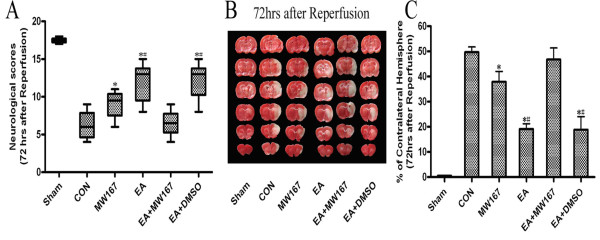
**Neurological scores (A) and infarct volumes (B, C) at 72 h after reperfusion in the rats.** Pretreatment with EA significantly improved the neurological scores and reduced the infarct volume while the γ-secretase inhibitor MW167 abolished the protective effect of EA pretreatment. Treatment with MW167 in MCAO only animals also induced neuroprotective effect. (*P < 0.05 vs. CON; #*P* < 0.05 vs. MW167).

All rats were sacrificed at 72 h after reperfusion for the measuring the infarct volumes (n = 8). EA pretreatment remarkably improved functional outcome as indicated by the significantly higher neurological function score [13.0(8–15) vs. 6.0(4–9), 72 h] and reduced infarct volume compared with CON group (19.1 ± 2.1% vs. 49.7 ± 2.2%) (*P* < 0.05, EA vs. CON). The infarct volume in EA + MW167 group was significantly larger than that in EA group (46.8 ± 4.6% vs. 19.1 ± 2.1%), and the neurological function score was significantly lower than that in EA group [6.0(4–9) vs. 13.0(8–15), 72 h](*P* < 0.05, EA + MW167 vs. EA). There were no statistical significance in the neurological function score and infarct volume between EA + DMSO and EA groups (*P* = 1.00; *P* = 0.98) or CON and EA + MW167 groups (*P* = 0.83; *P* = 0.58). Treatment with MW167 in MCAO only animals also resulted in reduced brain damage, which manifested as higher neurological function score and decreased infarct volume compared with CON group (*P < 0.05, MW167 vs. CON), but the cerebral injury of MW167 group was more severe than that of EA and EA + DMSO groups (#*P* < 0.05, MW167 vs. EA, MW167 vs. EA + DMSO) (Figure5).

## Discussion and conclusions

Our findings provide the first evidence that activation of Notch signaling contributes to EA pretreatment-induced cerebral ischemic tolerance. EA pretreatment before ischemia significantly increased Notch1,Notch4 receptors and Jag1 ligand mRNA in the striatum of adult rats and did not affect the Notch1 NICD and Hes1 mRNA levels, suggesting no activation of Notch pathway. However, after ischemia/reperfusion, Hes1 mRNA and Notch1 NICD protein level in EA group reached the peak at 2 h and 24 h, respectively, while both of them reached the peak at 72 h in CON group. The earlier activation of Notch signal may be caused by the higher expression of Notch1, Notch4 and Jag1 before ischemia in the EA pretreatment group. Although EA pretreatment can not increase the activation of Notch pathway before ischemia/reperfusion, it prepared the essential materials for the following Notch pathway activation after ischemia/reperfusion. Intraventricular injections with MW167 can block the cleavage of all four Notch receptors and lead to inactivation of Notch pathway in the central nervous system [12]. The γ-secretase inhibitors may prove to be more superior agents than that target a single cell type, because they may target pathogenic events in multiple cell types (neurons, lymphocytes and microglia) involved in cerebral ischemic injury. Our current data demonstrated that the neuroprotective effect of EA preconditioning was abrogated in the presence of intraventricular injections with MW167, suggesting that γ-secretase-mediated Notch signaling is required for the cerebral ischemic tolerance induced by EA pretreatment. Thus, the activated Notch signaling at the early stage after reperfusion is beneficial to the survival of neurons in the ischemic cerebral tissues.

Although the pivotal roles that Notch signaling takes part in the cellular differentiation of the developing central nervous system (CNS) have been proved, the functions of the signaling pathway, especially under pathological conditions in the adult CNS, remain to be illuminated. A line of data indicates that the expressions of notch receptors were up-regulated after MCAO in the brain of rat. [5,7,13]. The same phenomenon was observed in our study which demonstrated that focal cerebral ischemia/reperfusion can enhance Hes1 mRNA and Notch1 NICD protein level in striatum of adult rat. However, different to our results, Kawai et al. found that the mRNA of Hes5, an important downstream gene of Notch signal, was decreased on days 1 and 3 after global ischemia in rats, suggesting that the Notch signaling may be inhibited after transient forebrain ischemia [14]. The differences in animal strain, stroke model or treatment regimen may account for the discrepancy. So the phenomenon that ischemia-reperfusion can modulate Notch pathway in the CNS has been widely accepted.

In this study, we found that intraventricular injections with the γ-secretase inhibitor MW167 without EA pretreatment could reduce brain damage and ameliorated functional outcome in a rat model of stroke, suggesting that the inhibition of Notch signaling activation after ischemia/reperfusion could lessen the cerebral injury. In accordance with our results, it has been reported that Notch signaling increase the vulnerability of neurons to apoptosis. And γ-secretase inhibition improves functional outcome and reduces brain damage in ischemic stroke in mice by activating microglial cells and facilitating the infiltration of proinflammatory leukocytes [5]. However, some results support the idea that the activation of Notch pathway exerts the neuroprotective effect after ischemia. Gastric lavage with soybean isoflavone decreased the ischemia-induced apoptosis in the cerebral cortex and this effect of soybean isoflavone was associated with activation of the Notch signaling in the cerebral cortex [15]. Notch receptor activation inhibits cell death in neural stem cells *in vitro* rapidly, and this survival effect of Notch ligands was antagonized by a γ-secretase inhibitor. A more delayed activation of Notch signaling, which was induced by infusing the Notch ligand Dll4 into the cerebral lateral ventricle of adult rats, had no effect on improving cerebral infarct size but improved the motor skills over a period of 45 days [3]. A possible explanation for these apparently contradicting results is the different time frame. Our data showed that the earlier activation of Notch signal after ischemia/reperfusion induced by EA pretreatment provided protective effects.

However, our results raise the issue of why the earlier activation of Notch signaling induced by EA can change its characteristic from possible damaging effect to protective effect. The cerebral infarction induced by MCAO in rats and mice can be thought as a sphere in which cells in the inner core die rapidly by necrosis, in minutes to a few hours. However the cells in the outer “penumbral regions” die slowly, in a period of days to weeks. The evidence that neurons in the cerebral ischemic penumbra undergo apoptosis was first provided by Linnik and coworkers [16]. Whether or not a neuron survives when exposed to potentially lethal injury is likely to depend upon which of the antiapoptotic and proapoptotic pathways prevails. Studies of stroke in animal models support the hypothesis that the brain activates antiapoptotic signaling pathways involving neurotrophic factors and cytokines as responds to ischemic insults. The ischemia-related insults induce the rapid increased expression of several neurotrophic factors and cytokines in the rodent brain, leading to activation of signaling pathways that result in suppressing oxygen radical production and stabilizating cellular calcium homeostasis [17,18]. The effect of Notch pathway depends on the integration with other cellular signals, the degree of Notch activation, and the time point when the Notch pathway is activated during the cell cycle in development [19]. Earlier activation of Notch signaling in the striatum induced by EA pretreatment probably enhanced more survival signals. Later activation of Notch pathway resulted from ischemia/reperfusion in CON induced more neurodegenerative pathways.

Previous *in vitro* studies suggest that the cleavage of Notch activates the survival cascade downstream of the insulin receptor involving the serine/threonine kinase Akt and mammalian target of rapamycin, and that Ser727 phosphorylation of STAT3 also mediates the survival effects induced by Notch activation in neural stem cells [3]. There is a crucial physiologically crosstalk between the two major signal transduction pathways — Notch–Hes and JAK–STAT in the central nervous system [20]. It is demonstrated that the neuroprotective effect of EA pretreatment may be relevant to activation of PI3K/Akt and JAK/STAT signals [21,22]. These results imply that the activation of Notch signaling may link the EA pretreatment with some survival signal transducers. Notch pathway is also a useful paradigm to demonstrate the complexity of cross-talk pathway with nuclear factor-kB, transforming growth factor-β and Wnt signalings in ischemia/reperfusion [23,24].

In conclusion, the current study provides novel insights into the neuroprotection induced by EA pretreatment: EA preconditioning induces neuroprotection by controlling the timing of Notch pathway activation. However, further studies are demanded to delineate the downstream factors of Notch signaling for understanding the mechanisms of EA pretreatment-induced brain ischemia tolerance.

## Methods

### Instrumentation of animals

The experimental protocols were approved by the Ethics Committee for Animal Experimentation and were performed according to the Guidelines for Animal Experimentation of the Fourth Military Medical University. The animals were provided by the Experimental Animal Center of the Fourth Military Medical University. Adult male Sprague–Dawley (SD) rats weighing 280–320 g were housed under controlled condition with a 12 h light/dark cycle, temperature at 21°C ± 2°C, and humidity in 60%-70%. The rats were allowed to freely access to standard rodent diet and tap water.

### Experimental protocols

**Figure 6:**
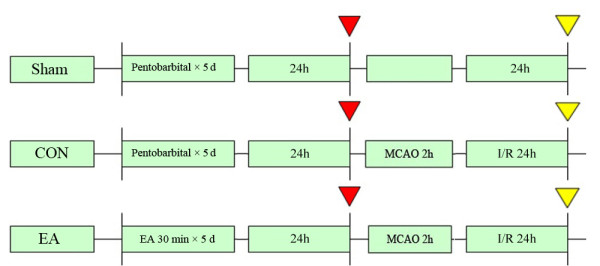
**Experimental protocol diagram 1.** Eighteen male SD rats were randomly divided into 3 groups(n = 6 each): Sham, CON, EA group. At 24 h after the last EA pretreatment (red arrow), the rats (n = 3) from 3 groups were killed for real-time RT-PCR analysis for Notch1, Notch4, Jag1 and Hes1 genes. At 24 h after reperfusion (yellow arrow), the rats (n = 3) from 3 groups were killed for double immunofluorescent staining to show the NICD immunoreactivity.

**Figure 7:**
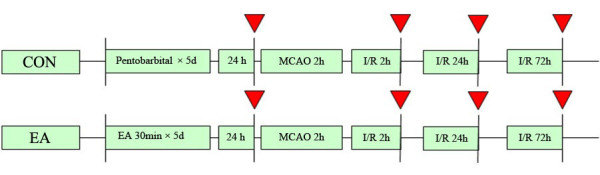
**Experimental protocol diagram 2.** Forty-eight male SD rats were randomly divided into 2 groups (n = 24 each): CON, EA group. At 24 h after the last EA pretreatment and 2 h, 24 h, 72 h after ischemia/reperfusion (red arrow), the rats (n = 6 for each time point) from 2 groups were anesthetized and killed. RT-PCR and Western blot analysis were performed.

**Figure 8:**
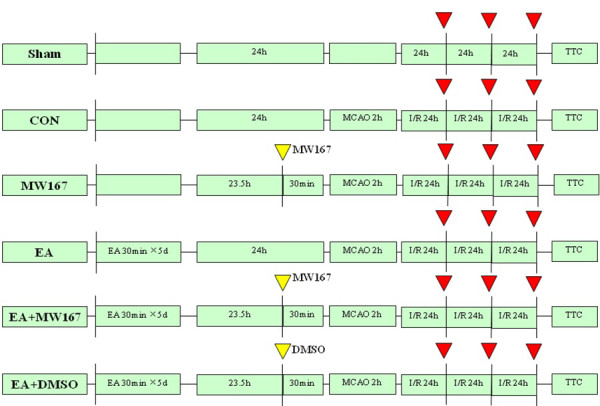
**Experimental protocol diagram 3.** Forty-eight male SD rats were randomly divided into 6 groups(n = 8 each): Sham, CON, EA, MW167, EA + MW167 and EA + DMSO group. At 30 min before MCAO(yellow arrow), intraventricular injections were performed. MW167 and DMSO were given respectively. At 24 h, 48 h and 72 h after ischemia/reperfusion (red arrow), neurological function scores were assessed in the 6 groups respectively. At 72 h after ischemia/reperfusion, all rats were anesthetized and killed, the infarct volumes were measured.

Eighteen male SD rats were randomly divided into 3 groups(n = 6 each): Sham, CON, EA group. Sham and CON groups received 40 mg/kg SP for 5 days, while EA group received both 40 mg/kg SP and 30 min EA stimuli for 5 days. At 24 h after the last EA pretreatment (red arrow), the rats (n = 3) from 3 groups were killed for real-time RT-PCR analysis for Notch1, Notch4, Jag1 and Hes1 genes. At 24 h after reperfusion (yellow arrow), the rats (n = 3) from 3 groups were killed for double immunofluorescent staining to show the NICD immunoreactivity (Figure6). Forty-eight male SD rats were randomly divided into 2 groups (n = 24 each): CON, EA group. At 24 h after the last EA pretreatment and 2 h, 24 h, 72 h after ischemia/reperfusion (red arrow), the rats (n = 6 for each time point) from 2 groups were anesthetized and killed. RT-PCR and western blot analysis were performed (Figure7). Forty-eight male SD rats were randomly divided into 6 groups(n = 8 each): Sham group (Sham),Control group (CON),Electroacupuncture group (EA), γ-secretase inhibitor MW167 group (MW167),EA + MW167 group (EA + MW167),and EA + Vehicle group (EA + DMSO). Rats in Sham group did not receive any treatment only given the same surgical procedure without arterial occlusion. Animals in other 5 groups were subjected to middle cerebral arterial occlusion (MCAO). Rats in CON and MW167 groups were anesthetized and inhaled oxygen for successive 5 days as the EA group, but received no EA pretreatment. Animals in the EA,EA + MW167 and EA + DMSO groups received the same EA pretreatment. Intraventricular injections were performed in MW167, EA + MW167 and EA + DMSO groups (Figure8).

### Electroacupuncture pretreatment and transient focal cerebral ischemia

EA pretreatment and transient focal cerebral ischemia were performed as described previously [10]. Briefly, rats were anesthetized with sodium pentobarbital (40 mg/kg, i.p.) and inhaled oxygen by face mask at a flow rate of 1 L/min. The Baihui acupoint (GV20), which is located at the intersection of the sagittal midline and the line linking two ears, was stimulated with the intensity of 1 mA and frequency of 2/15 Hz for 30 min per day for successive 5 days by using the G6805–2 EA Instrument (Model No.227033; Qingdao Xinsheng Ltd). A transient right middle cerebral artery occlusion (MCAO) was performed at 24 h after the last pretreatment. Reperfusion was accomplished by withdrawing the suture after 120-min ischemia. Blood flow through the middle cerebral artery was measured by laser Doppler flowmetry (PeriFlux 5000, Perimed AB, Sweden). Rats retaining >20% of baseline perfusion during ischemia were excluded. The incision sites were infiltrated with 0.25% bupivacaine hydrochloride. Temporalis muscle temperature was monitored and maintained at 37.0°C-37.5°C by surface heating or cooling during surgery until rats recovered from anesthesia.

### Intraventricular injections

Intraventricular injections were performed at 30 min before MCAO. Animals were anesthetized intraperitoneally with sodium pentobarbital, then MW167 (Merck) was injected unilaterally into the right brain lateral ventricle with a 32-gauge needle attached to a Hamilton syringe mounted on a stereotactic device (Kopf Instruments, Inc., Tujunga, CA). Stereotactic coordinates were as follows: A, -0.1, R, 1.5, and H, 3.8 (coordinates corresponding to the right lateral ventricle) [25]. MW167 was dissolved in PBS with 10% DMSO and used at the concentration of 1 mM [12]. Vehicle (10% DMSO + PBS) was used as a control treatment. The MW167 solution and the vehicle were injected over 5 min in a volume of 12 μl.

### Real-time PCR

Three rats per group were killed with an overdose i.p. injection of pentobarbitone (100 mg/kg body weight). Hippocampus and striatum were harvested and total RNA was isolated with RNAiso Plus (TaKaRa), according to a standard protocol. Quantitative reverse transcription polymerase chain reaction (RT-PCR) was performed with the SYBR green real-time PCR method on an IQ5 RT-PCR instrument (BIO-RAD) with 3-stage program parameters provided by the manufacturer. Each sample was tested in triplicate, and samples were obtained from 3 independent experiments that were used for analysis of relative gene expression data with the 2^-ΔΔCT^ method. The following primers for RT-PCR were designed by TaKaRa corporation : Notch1: (Fwd: AAT GGA GGG AGG TGC GAA G, Rev: ATG GTG TGC TGA GGC AAG G); Notch4: (Fwd: CCT GGA CAG CAA TGC CAA GA, Rev: AGT CCA GCC CTC GTT ACA CAC AC); Jag1: (Fwd: CCA GCG GTC CTA ATG GTG ATG, Rev: GCT GTG GTT CTG AGC TGC AAA G); Hes1: (Fwd: TGT CAA CAC GAC ACC GGA CA, Rev: GCC TCT TCT CCA TGA TAG GCT TTG); GAPDH: (Fwd: GGC ACA GTC AAG GCT GAG AAT G, Rev: ATG GTG GTG AAG ACG CCA GTA).

### Western blot

To test Notch1 NICD level, three rats per group were killed with an overdose i.p. injection of pentobarbitone (100 mg/kg body weight). The striatum at bregma −2.0 to 2 mm was removed before MCAO and at 2 h, 24 h, or 72 h after reperfusion and homogenized with total protein extraction kit (KeyGEN, Nanjing, China) on ice. The following primary antibodies were used in this study: rabbit anti-Notch1 NICD (1:400; Abcam) or mouse anti-β-actin (1: 1000; Santa Cruz Biotechnology). Secondary horseradish peroxidase–conjugated goat–anti-rabbit antibody for NICD or goat–anti-mouse antibody for β-actin (Pierce Biotechnology Inc; 1:5000 dilution) were used. Quantitative analysis for immunoblotting was performed after scanning of the X-ray film with a Quantitative-One software (version 4.60, Bio-Rad, USA). The changes in relative content of NICD expression were represented as the ratio of integrated optical density of the band of NICD to that of β-actin.

### Double immunofluorescence staining

At 24 h after reperfusion, three rats from each of the Sham, CON and EA groups were fixed by transcardial perfusion with saline followed by perfusion and immersion in 4% paraformaldehyde. A series of adjacent coronal 10 μm-thick sections were cut from the blocks (bregma A = −2.0-2.0 mm). NICD/NeuN double immunofluorescence analyses were performed. A monoclonal antibody against NeuN (1:1000, Millipore) and a polyclonal antibody against Notch1 NICD (1:400, Abcam) were used. Fluorescein isothiocyanate (FITC, Calbiochem) and cyanine-5.18 (CY5, Jackson Immunoresearch) were used for double-label immunostaining.

The total numbers of NICD- and NeuN-positive cells in five separate fields of each section from the ipsilateral striatum were counted with use of a microscope (Olympus, BX51, Japan) (40×). The values were averaged and expressed as mean number of NICD- and NeuN-positive cells per field in striatum.

### Neurobehavioral evaluation and infarct assessment

At 24, 48, 72 h after reperfusion, an 18-point scoring system reported by Garcia et al. [26] with modifications was used for neurological assessment by a blinded observer. Then, rats (n = 8 for each group) were decapitated and 2-mm thick coronal sections throughout the brain were stained with 2% 2,3,5-triphenyltetrazolium chloride (Sigma-Aldrich) to evaluate the infarct volume, as described previously [10].

### Statistical analysis

Brain sections were examined by two independent and blinded investigators. The software, SPSS 11.0 for Windows (SPSS Inc., Chicago, IL), was used to conduct statistical analyses. All values, except for neurological function scores, are presented as mean ± SD. The infarct volumes, Notch1 NICD level and Notch1, Notch4, Jag1 and Hes1 mRNA level were analyzed by one-way analysis of variance, and differences between-groups were detected with *post hoc* Student–Newman–Keuls test. The neurological function scores were expressed as median (range) and were analyzed with Kruskal–Wallis test followed by Mann–Whitney *U* test with Bonferroni correction. Values of *P*<0.05 were considered as statistically significant.

## Misc

Yu Zhao and Xiyao Chen contributed equally to this work.

## Competing interests

The authors declare that they have no competing interests.

## Authors’ contributions

ZZ, QW, LX, JP and SC conceived the study, participated in the experiment design and drafted the manuscript. YZ, XC, LM, XZ and EH performed the experiments and analysed and interpreted the data. LH, ZZ, QW and YZ participated in the data analysis, were involved in drafting the manuscript, and made important intellectual contributions. All authors read and approved the final manuscript.
